# DNA methylation topology differentiates between normal and malignant in cell models, resected human tissues, and exfoliated sputum cells of lung epithelium

**DOI:** 10.3389/fonc.2022.991120

**Published:** 2022-10-27

**Authors:** Jian Tajbakhsh, Fariborz Mortazavi, Nirdesh K. Gupta

**Affiliations:** ^1^ Department of Surgery, Cedars-Sinai, Los Angeles, CA, United States; ^2^ Samuel Oschin Comprehensive Cancer Institute, Cedars-Sinai, Los Angeles, CA, United States; ^3^ 3rd Street Diagnostics, Cedars-Sinai, Los Angeles, CA, United States; ^4^ Division of Hematology/Oncology, West Los Angeles Veterans Affairs (VA) Medical Center, Los Angeles, CA, United States; ^5^ Department of Medicine, University of California, Los Angeles, CA, United States

**Keywords:** global DNA methylation, 5-methylcytosine, sputum, lung cancer, confocal microscopy, 3D image cytometry, single-cell analysis, nuclear topology

## Abstract

**Background:**

Global DNA hypomethylation is a prominent feature of cancer cells including lung cancer, that has not been widely explored towards cancer diagnosis. In this study we assess the comparative distribution of global DNA methylation in normal cells versus cancer cells in various specimen models.

**Methods:**

We used *in situ* immunofluorescence labeling of overall 5-methylcytosine (5mC) and covisualization of global DNA (gDNA) by 4’,6-diamidino-2-phenylindole (DAPI), confocal microscopy and 3D image analysis to derive 5mC/DAPI colocalization patterns in human cell lines (BEAS-2B, A549, H157) and upper respiratory epithelial cells derived from various sources (i.e., sputum from healthy and cancer patients, and resected tissues from normal parenchyma and lung tumors).

**Results:**

By introducing 5mC/DAPI colocalization index as a metric we could distinguish between normal epithelial cells and aberrantly hypomethylated cancer cells. Cultured lung cancer cells (H157 and A549) had significantly lower indices compared to normal cells (BEAS-2B). Furthermore, we were able to identify such extensively hypomethylated low-index cells in tumor tissues and the matching sputum from cancer patients. In contrast, the indices of cells derived from sputum of healthy individuals had more similarity to epithelial cells of normal parenchyma and the phenotypically normal BEAS-2B cells.

**Conclusions:**

The results suggest that 5mC topology using high-resolution image cytometry shows potential for identifying hypomethylated cancerous cells in human tissues and amongst normal cells in matching sputum, which may render a valuable surrogate for biopsied tissues. This promising feature deserves further validation in more comprehensive studies.

## Introduction

In cancer cells two types of aberrant DNA methylation features coexist: 1) promoter hypermethylation of a few genes and 2) global hypomethylation, mostly attributed to severe hypomethylation of repeat sequences that comprise more than two-thirds of the human genome (1–3). The most prevalent types of repetitive sequences include long interspersed nucleotide element 1 (LINE-1) and *Alu* sequences that contribute to around 30% of the genome. The analysis of DNA hypomethylation has been largely remained unexploited although it has been known for decades that global hypomethylation is prevalent in tumors compared to normal cells (4, 5). Generally, cancer cells show a net hypomethylation, containing 20–60% less genomic 5mC than their normal counterparts. Global DNA hypomethylation occurs in many of the major cancer types, including the tumors of the breast, colon, head and neck, bladder, esophagus, liver, prostate, stomach, and lung (6, 7). Thus, the global methylation status is a unique feature of cells and tissues and global hypomethylation is a common epigenetic process in cancer, which may progressively evolve during multistage carcinogenesis.

Because of its high frequency in the genome, methylation in LINE-1 has shown to be a good indicator of the global DNA methylation level within a cell (7–9). LINE-1 is heavily methylated in normal human tissues. Hypomethylation of LINE-1 repetitive elements has been described as one of the key hallmarks of tumorigenesis. This shift was also shown in lung tumor cells (7, 10–12) and in blood cells of lung cancer patients (13, 14). Moreover, the degree of LINE-1 hypomethylation is associated with clinical data and survival prognosis (8, 15). The selection of literature amongst numerous other publications are a proof of evidence that global hypomethylation largely exists in both subtypes of non-small cell lung cancer (NSCLC), squamous cell carcinoma (SCC) as well as in adenocarcinoma. Chalitchagorn et al. (7) evaluated the differential level of LINE-1 methylation between normal tissues and cancers in a broad panel of malignancies including NSCLC (7). The investigators detected significantly greater hypomethylation in most cancers including carcinomas of the lung. Daskalos et al. (2009) reported that LINE-1 and Alu methylation indices in primary tumors strongly correlated with each other (12). However, clinicopathological parameters such as age, gender, T status (size and extension of the tumor), differentiation and nodal metastasis did not correlate with LINE-1 and *Alu* methylation. Notably LINE-1 hypomethylation was found more frequent in SSC than in adenocarcinoma, however only at borderline significance (p = 0.052). Suzuki et al. (2013) disclosed that through accurate measurement of methylation levels using pyrosequencing, hypomethylation of LINE-1 was frequently detected in NSCLC and associated with various clinical features (11). Tumor tissues showed significantly lower levels of LINE-1 methylation when compared with matched nonmalignant lung tissues. A Study by Ikeda et al. (2013), also using pyrosequencing, revealed that methylation levels of resected lung cancer tissue were significantly lower than that of matched normal lung tissues (8). The association between clinical characteristics and methylation levels of lung adenocarcinoma tissues has revealed that higher histologic grade and positive findings for vascular invasion were significantly associated with stronger hypomethylation. Furthermore, previous studies brought to light that hypomethylation is related to worse prognosis of NSCLC, that is significantly shorter disease-free intervals after curative resection. Along the same lines, the methylation rate by LINE-1 contribution was significantly lower in squamous cell carcinoma than in adenocarcinoma (11, 15).

To date, differential DNA methylation analysis has been quantitatively assessed mostly by molecular approaches including electrophoretic, chromatographic, polymerase chain reaction (PCR) based, array based, and sequencing technologies (16, 17). Despite tremendous improvement in specificity, sensitivity, and the inherent single-base resolution of these methods, they remain technically and economically challenging in the high-throughput analysis of single cells (18). These include the limitation of PCR-based approaches in multiplexing and the challenging sensitivity and cost issues of whole-genome sequencing, especially for the interrogation of repetitive elements. Considering the prevalence and load of DNA methylation imbalances —especially hypomethylation of repeat sequences— imaging-based assessment of changes in global nuclear 5mC patterns provides a powerful alternative for the massively parallel analysis of cells. The reason being that DNA hypomethylation at such scales involves large-scale chromatin reorganization that can be made visible by light microscopy (19–21). Beyond *in situ* methods, the dynamics of global DNA methylation has been successfully visualized by live-cell imaging using constructed reporters (22–24).

We had previously introduced an image-cytometric approach termed three-dimensional quantitative DNA methylation imaging (3D-qDMI), a nondestructive *in situ* method that entails the parallel quantitative measurement of 5mC load and spatial nuclear distribution to be used for the characterization of cells and tissues (25, 26). 3D-qDMI combines immunofluorescence, high-resolution confocal microscopy and 3D image analysis, and allows for the rapid, parallel, single-cell phenotyping of thousands of cells within heterogeneous samples. 3D-qDMI has been successfully applied in the characterization of mouse and human cells and tissues of various origin (27–33). Utilizing this high-content tool, in this study we explored the comparative 5mC topology in normal epithelial and cancer cell models of the lung, as well as in cells of resected human tissues and exfoliated upper respiratory epithelial cells derived from matching sputum samples from cancer patients and healthy donors.

## Materials and methods

### Cultured cells

A549 and H157 cells (ATCC) were routinely cultured in RPMI supplemented with antibiotics and 10% heat-inactivated fetal bovine serum (FBS) (Omega Scientific). BEAS-2B cells (ATCC) were cultured in bronchial epithelial cell basal medium (BEBM) supplemented with all 1x BEGM (bronchial epithelial growth medium) SingleQuots kit additives (all from Lonza): 2ml of bovine pituitary extract (BPE), 0.5 ml of hydrocortisone, 0.5 ml of human epidermal growth factor (hEGF), 0.5 ml of epinephrine, 0.5 ml of transferrin, 0.5 ml of insulin, 0.5 ml of retinoic acid, triiodothyronine, and 0.5 ml gentamicin/gentamicin-B. According to the manufacturer’s protocol to formulate 500 ml of BEGM, the entire volume of each additive in the kit was added to 500 ml of BEBM. All cells were grown to 70% confluency in 5% CO_2_ at 37°C.

### Patient materials

The study utilized pre-surgical sputum samples and post-surgical tissue from three patients with stage I-II NSCLC. Sputum samples were collected following obtaining the patients’ written consents. Surgically resected specimens were provided in a deidentified manner and were exempt from patients’ consents. Since this study was categorized as basic research, information that could lead to patient identity and patient characteristics such as age, gender, and clinical information were masked, and each sample received a research code.

### Sputum collection and processing

Patients were given a cup of water to gurgle, immediately before sputum induction, to minimize the contribution of oral cavity saliva in the sample. The actual sputum induction was performed by inhalation of hypertonic saline (NaCl 4.5%). Aerosols were generated by an ultrasonic nebulizer, with an output at 1.5 mL/min. The subjects inhaled the saline solution aerosols for a fixed period of 15 min. Then subjects were encouraged to expectorate sputum. Samples were collected in a plastic container and kept at 2–8°C until processing for cell extraction. For purification of cellular components from mucus and other contaminants, samples were processed twice as follows. Samples were diluted with phosphate-buffered saline solution (PBS) containing 10 mM dithiothreitol (DTT), then centrifuged at 400g for 10 min at 4°C to separate cellular and fluid phases. The ultimate cell pellet was resuspended in phosphate-buffered saline (PBS) containing 20 mM ethylenediamine tetra-acetic acid (EDTA) and 2% human serum albumin. Cell counts were performed on centrifuged samples and a sample of about 5x10^5^ cells was spread on a microscope slide. Subsequently, cells were fixed in 4% paraformaldehyde for 15 minutes. Then the slide was kept in PBS at 2–8°C until further processing for immunofluorescence.

### Immunofluorescence assay

Immunofluorescence staining was performed in four-chamber microplates (ThermoFisher Scientific) according to previously established protocols (25, 28, 33). The primary and secondary antibody set included unconjugated mouse anti-5-methylcytosine monoclonal antibody (AMM99021, Aviva Systems Biology) at 1 mg/ml and Alexa488-linked donkey anti-mouse IgG (A-21202, ThermoFisher Scientific) at 5 mg/ml final concentrations. The cells were subsequently delineated with the cytoplasmic marker Cell Mask Red (ThermoFischer Scientific) and cell nuclei counterstained with DAPI. The specificity/dynamic range of the anti-5mC antibody was tested as previously reported in (29) (data not shown in here). Formalin-fixed tissue sections at 5 µm thickness were kept floating in 10% formalin at 2−8°C until immunofluorescence staining. Prior to staining, tissues were transferred to microwell plates, washed in PBS at room temperature, then stained as floating tissues using the same protocol that was applied to fixed cells.

### Confocal imaging and 3D image analysis

Confocal imaging of labeled slides was performed using a TCS SP5 X Supercontinuum microscope (Leica Microsystems, Mannheim Germany). The system provides full freedom and flexibility in excitation and emission within the continuous range of 470 to 670 nm within 1 nm increment. The TCS SP5 X system was coupled with a 405nm diode laser line for excitation of DAPI fluorescence. Serial optical sections were collected at increments of 250–500 nm with a Plan-Apo 63×1.3 glycerol immersion lens. The pinhole size was consistently 1.0 airy unit. The output file format was a series of TIFF images that were utilized for 3D-image analysis. The typical image size was 1576×1576 with a respective voxel size of 189 nm ×189 nm × 500 nm (x, y, and z axes), and a dynamic intensity range of 12 bits per pixel in all four channels. All biomarker signals from optical sections were recorded into separate channels. All images were acquired under nearly identical conditions and modality settings. The drift of the settings during acquisition was considered minimal and therefore neglected. 3D image analysis was performed using a dedicated algorithm for multi-parametric high-content analysis, as previously described in (25, 26, 28). This image analysis tool operates in three steps. First cells (within imaged populations) were processed for 3D segmentation. Then fluorescence 5mC and DAPI signals were recorded for each cell nucleus, and subsequently two parameters were generated per cell: (a) the global 5mC intensity of the entire nucleus, and (b) the codistribution (2D scatter plots) of 5mC signals and gDNA (DAPI) signals. The angle under the regression line of the 5mC/DAPI codistribition plot is automatically calculated as the second parameter. The two parameters were output as DAT files. The results can be traced back for each imaged epithelial cell through a numerical identifier that was generated by the software. In addition, the software also calculated the similarity of 5mC/DAPI codistribution patterns between cells based on Kullback-Leibler (K-L) divergence and generated similarity maps of cells as previously described (25, 26). The pseudo-colors within a similarity map represent the four K-L characteristics known as soft-qualifiers: green (similar), blue (likely similar), yellow (unlikely similar), and red (dissimilar). The soft-qualifiers for each cell were generated through comparison of the 5mC/DAPI distribution pattern of the individual cell with the composite 5mC/DAPI pattern of the entire imaged cell population.

## Results

### Nuclear DNA methylation patterns distinguish between normal and transformed cells

The notion of our study was to explore differential three-dimensional (3D) nuclear 5mC patterns in normal versus cancerous human upper respiratory cells. To establish these differences, we started with three cultured human cell lines comprising the immortalized normal human epithelial cell line BEAS-2B, and the two NSCLC cell lines A549 (alveolar basal epithelial cells) and H157 (highly invasive lung carcinoma cells). BEAS-2B are epithelial cells that were established through isolation from normal human bronchial epithelium, obtained from autopsy of a noncancerous individual (34). These cells have been widely used as an *in vitro* cell model representing normal lung epithelial cells in a large variety of studies associated with respiratory diseases including lung carcinogenesis. A549 cells are adenocarcinoma human alveolar basal epithelial cells established through an explant culture of adenocarcinoma lung tissue of a 58-year-old Caucasian male (35). This cell line is categorized as NSCLC. On that note, NSCLC accounts for 85-88% of all cases of lung cancer. The A549 cell line is widely used as a model of lung adenocarcinoma, as well as an *in vitro* model for type II pulmonary epithelial cells (35). The more aggressive H157 cell line was established by A.F. Gazdar, H.K. Oie, J.D. Minna and associates in 1979 from a SCC of the buccal mucosa, from cells recovered from pleural effusion obtained from a patient prior to therapy.

**Figure 1:**
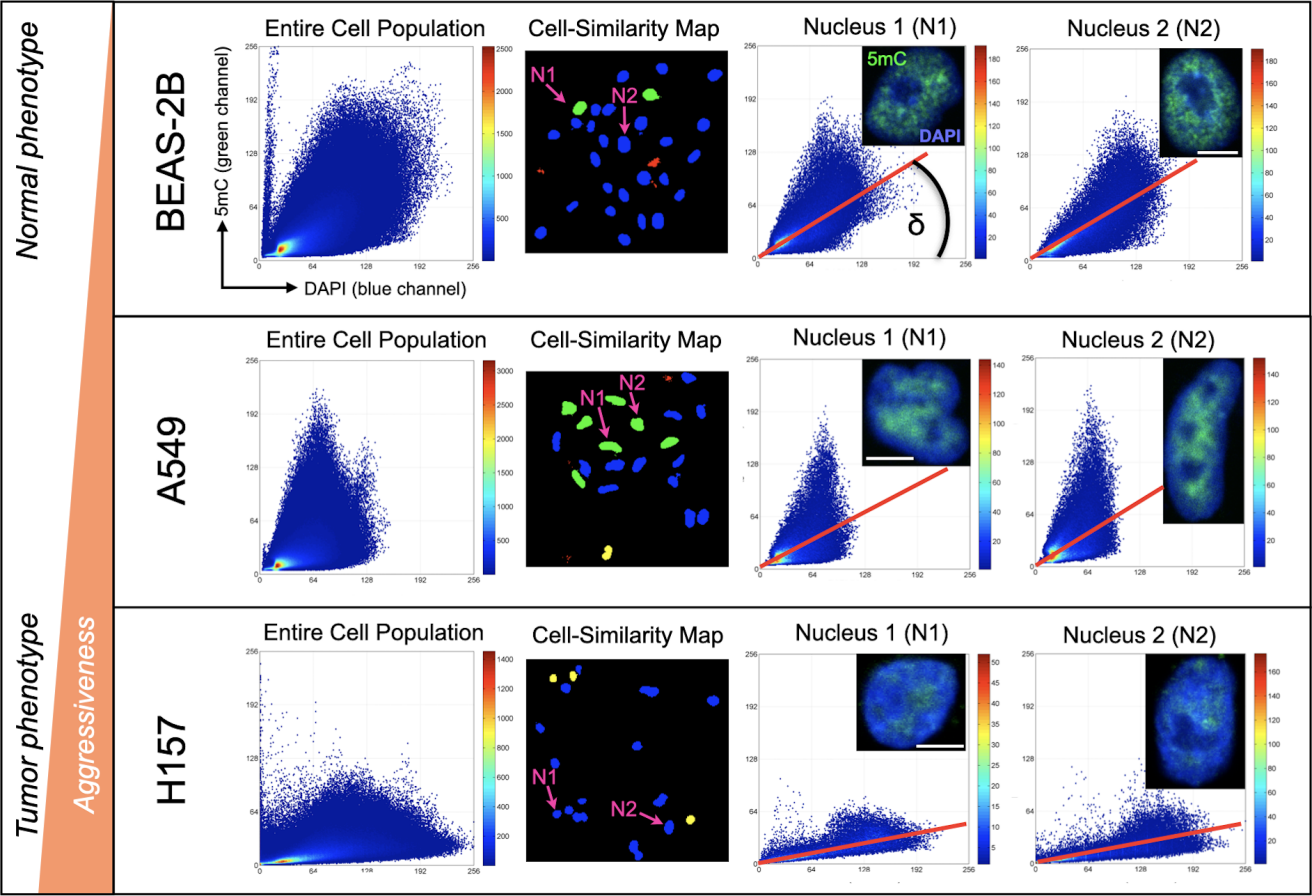
Global DNA methylation phenotyping of cultured cells with 3D-qDMI. The method was able to distinguish between the different cell types based on differential 5mC/DAPI distribution patterns calculated and displayed as individual heat map scatter plots (DAPI = x-axis, 5mC = y-axis). Plots were generated for the entire cell population (composite 5mC/DAPI codistribution of all measured cell nuclei) as the reference plot, and for each cell nucleus. Cell-similarity maps based on K-L divergence indicate a high degree of homogeneity in 5mC/DAPI topology for all three cell lines. This is due to most cells being similar (green nuclei) and likely similar (blue nuclei). Selected cell nuclei N1 and N2 for each cell line selected from the cell-similarity maps represent the most dominant 5mC topology for each cell line. The angle δ under the regression line (false-colored red) equals the 5mC/DAPI colocalization index for each nucleus. White bars are 5 µm.

Using K-L divergence, cell-similarity maps of 5mC/DAPI codistribution were generated as described in (25). Basically, each cell nucleus receives a numerical score which is converted into the four classifiers “similar”, “likely similar”, “unlikely similar” and “dissimilar”, with respective color-codes as explained in the Methods section. For clarity, the cell-similarity maps only contain cell nuclei extracted from fluorescence images (by our image analysis software). The more a cell population contains similar and likely similar cells the more homogenous it is regarding the cells’ 5mC/DAPI codistribution. We observed a high degree of homogeneity in 5mC patterns among cells for all three cell lines. This observation agrees with the fact that cultured cells typically align with the more dominant cell features, as represented by similar and likely similar cells in **Figure 1**. Thus, we conclude that the observed patterns are in fact dominant patterns for each cell line. **Figure 1** depicts 5mC/DAPI codistribution patterns for each of the three cell lines as scatter plots on the cell-population level and for individual representative cell nuclei (N1 and N2). Because of the high degree in feature homogeneity within a cell line, N1 and N2 are very similar in 5mC topology across all cell lines. We observed common global DNA methylation patterns amongst healthy cells that significantly differ from the 5mC/DAPI patterns of cancerous cells. We introduced the angle δ under the regression line of the plot as a measurable descriptor of 5mC/DAPI codistribution for each cell type, calling it the 5mC/DAPI codistribution index or simply the 5mC/DAPI index. This index is differential between the cell lines and corresponds to the aggressiveness of the cells. NSCLC cell line A549 displays slightly reduced (~20% on average) and H157 significantly reduced (> than 60% on average) 5mC loads compared to BEAS-2B cells. The same proportional reduction could be experienced for 5mC/DAPI colocalization indices. H157 cells, which are reported to have more metastatic potential than A549 cells, are even more hypomethylated displaying a flatter curve and in conjunction a smaller 5mC/DAPI index. These features indicate that a large portion of the highly condensed repeat sequences in the nuclei (as represented by DAPI-staining) is hypomethylated. From these initial results we glean that the method was able to successfully distinguish between the different cell types, specifically between the more normal and transformed phenotypes and in correlation with aggressiveness, based on differential 5mC/DAPI codistribution patterns (scatter plots).

### Normal and malignant tissue cells display differential 5mC phenotypes found in cultured cells

**Figure 2:**
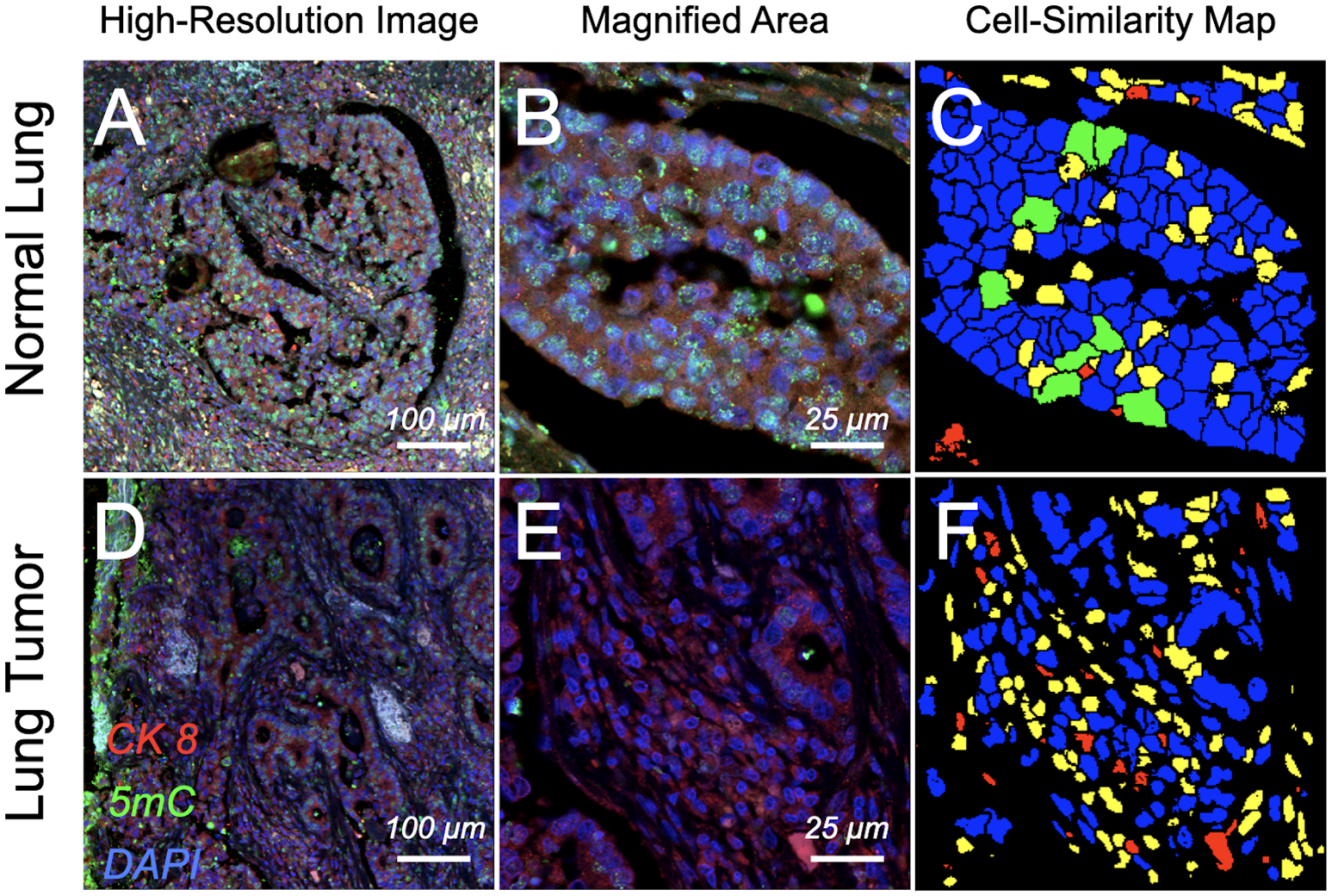
Confocal images of immunofluorescence-labeled lung cancer and adjacent normal tissue section. Surgically resected normal parenchyma and tumoral regions from patients that were diagnosed with lung cancer were *in situ* labeled for differential patterns of global DNA methylation. Cell nuclei containing global DNA marked by DAPI (false-colored blue) in normal lobules **(A)** and a magnified subarea on the same section **(B)** show higher degree of DNA methylation (5mC, false-colored green) compared with severely hypomethylated nuclei in ductal regions of the tumor **(D)** and a respectively magnified subarea **(E)**; cytokeratin 8 (CK 8) (red) was used as a marker to delineate the epithelial compartments. Cell-similarity maps **(C, F)** illustrate higher heterogeneity in cell composition for the tumor area compared to normal tissue, illustrated by the higher number of unlikely similar (yellow) and dissimilar (red) cells. In comparison normal tissue is populated by a majority of similar (green) and likely similar (blue) cells, thus presenting a high degree of cellular homogeneity.

**Figure 3:**
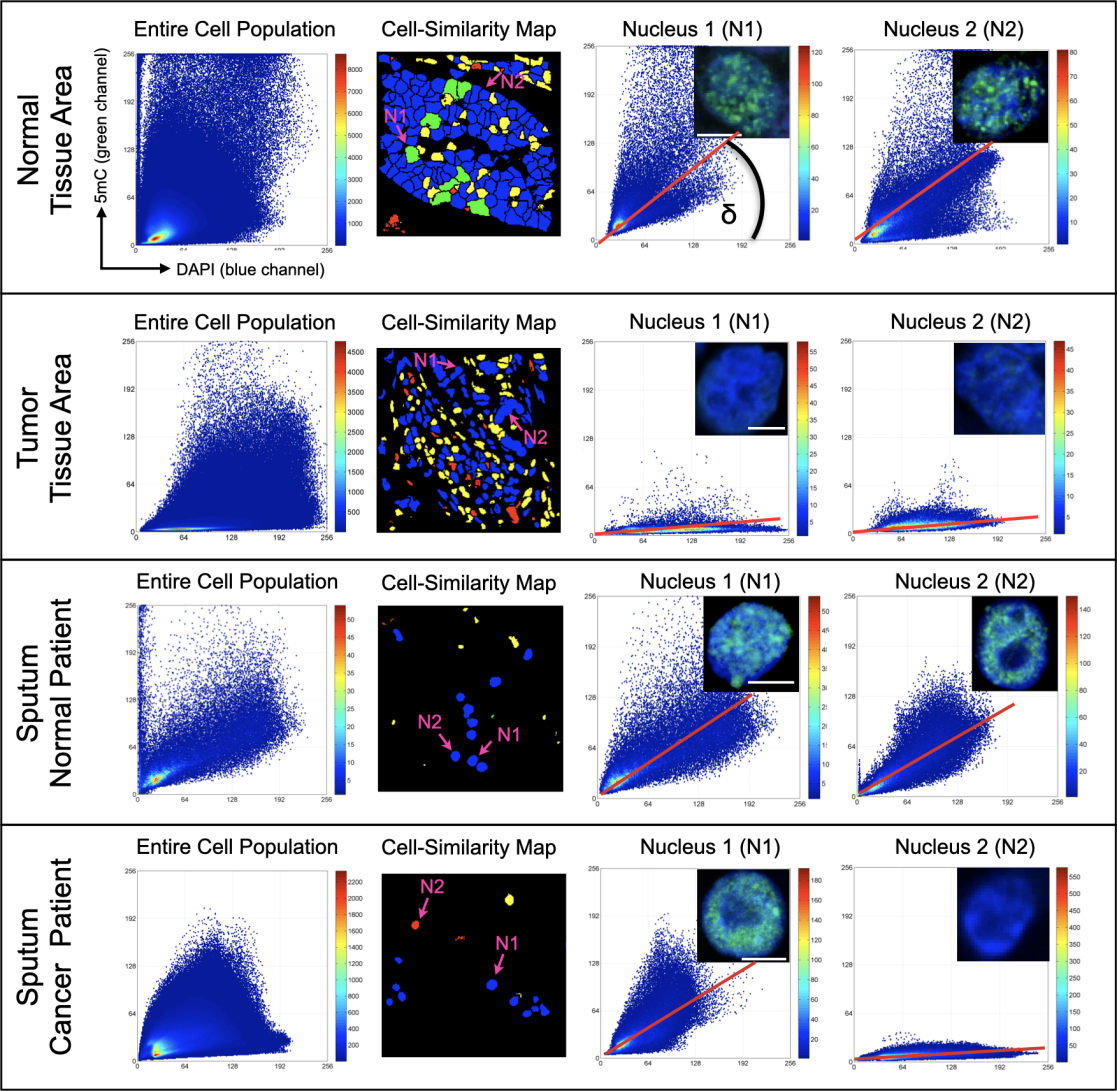
Comparative DNA methylation phenotyping of patient tissues and matching sputum. The normal lung epithelium as well as the normal sputum contain a majority of epithelial cells that display nuclear 5mC features with a high degree of similarity to the patterns seen in BEAS-2B cells. The two cell sources appear quite homogeneous in terms of 5mC topology. In contrast, the tumoral region appears more heterogeneous and contains a majority of severely hypomethylated cells. The selected nuclei N1 and N2 in the first three respective panels represent the most prevalent 5mC topology for the normal tissue and sputum as well as for the tumor area. The sputum from cancer patients contained severely hypomethylated cells amongst normally methylated cells. For this case the selected nuclei differ in 5mC indices: cell nucleus N1 represents the normally methylated cell whereas N2 constitutes an aberrantly hypomethylated cell. White bars are 5 µm.

Next, we tried to verify the observed 5mC patterns in surgically removed tissues from lung cancer patients and adjacent normal lung tissue, as well as in cells from matching sputum of the lung cancer patients versus sputum cells of the healthy donors (with no history of cancer). **Figure 2** shows a similar differential 5mC load in the fluorescently labeled section of normal parenchyma and from surgically resected lung tumor: a substantial degree of hypomethylation was seen by eye under the microscope in the nuclei of epithelial cells residing within the tumor area compared to their normal counterparts. A confirmation of the differential 5mC phenotypes was obtained using 3D image analysis. The same comparative relation as for the cell lines could be found in tumor tissue from lung cancer patients and in adjacent normal lung tissue: the normal lung section was populated by an absolute majority of epithelial cells with normal methylation patterns (5mC loads) and high 5mC/DAPI colocalization indices (**Figure 3**). In stark contrast, the cancerous tissue showed most cells having drastically reduced 5mC signal and significantly lower 5mC/DAPI indices. Again, in **Figure 3**, for the displayed tissue sections we selected only nuclei (N1 and N2) of cells that represented the most prevalent 5mC features within the epithelial compartment: that is either similar (blue) or likely similar (green) nuclei.

### Sputum of cancer patients contain cells resembling malignant 5mC features

Notably, using 3D-qDMI the same differential 5mC phenotypes were also present in cells from matching sputum of the lung cancer patients versus sputum cells of the healthy donors (with no history of cancer) (**Figure 3**). In the sputum from normal (healthy) donors we only found nuclei of epithelial cells with high resemblance to the most dominant cytometric 5mC/DAPI indices found in BEAS-2B cells and cells in the phenotypically normal area of the lung tissue section. Thus, the selected representing nuclei N1 and N2 are very similar in their 5mC features. In comparison, the sputum from cancer patients contained severely hypomethylated cells amongst normally methylated cells. For this case **Figure 3** is showing the selected cell nuclei that differ in 5mC/DAPI indices: N1 represents the normally methylated cell and N2 constitutes an aberrantly hypomethylated cell. Please note that the displayed high-resolution images only exhibit a portion of the whole slide. The aberrant cells in the cancer sputum share a high likeliness in 5mC/DAPI indices with hypomethylated tumor cells in biopsied cancer tissues and the more aggressive H157 cell line (with higher metastatic potential). These sputum cells are characterized by an extremely flat regression line (δ < 15°).

## Discussion

Measuring alterations in DNA methylation is a valuable method for detecting cancer cells. This phenomenon correlates with early events in carcinogenesis and tumor progression and can serve as a signature in early cancer detection and for therapeutic monitoring.

Cytometric analysis of global DNA in conjunction with cell imaging was introduced for tissue characterization towards the end of the 1990ies but did not gain much popularity in comparison to contemporaneously developed molecular methods. These methods are based on different platforms such as high-pressure liquid chromatography (HPLC), liquid chromatography coupled with tandem mass spectrometry (LC-MS/MS), the more recent capillary electrophoresis (CE), and more simplified assays: such as the luminometric methylation assay (LUMA), enzyme-linked immunosorbent assay (ELISA) based assays, as well as pyrosequencing and the real-time PCR based MethyLight (9, 36–43). The latter two methods approximate global DNA methylation by quantifying LINE-1 and *Alu* DNA.

Nevertheless, cytometric analyses such as image cytometry and flow cytometry have the advantage of being nondestructive and requiring no error-prone DNA extraction steps, while providing information at single-cell resolution in a highly paralleled and throughput fashion. However, image cytometry was applied in combination with radio-labeled or enzymatic reporters for detection, which either lack sensitivity, multiplexing capability or affect repeatability (consistency) of the assay, and failed to provide enough significance in differential results due to low image resolution. Furthermore, flow-cytometry provides either average 5mC measurements across a large population of cells or only quantifies mean 5mC intensity values in cell nuclei. In the meantime, molecular methods have advanced to also measure analyte contents at single-cell resolution. Yet, both technologies still lack to produce the quality of information that *in situ* methods yield regarding subcellular target localization and distribution.

In contrast to these methods including previous cell imaging-based attempts, 3D-qDMI leverages the extraction of differential 5mC topology by considering secondary effects of DNA methylation imbalances that occur throughout cellular transformation, especially hypomethylation of global DNA. In particular, the latter mechanism elicits reorganization of the genome within cell nuclei, affecting nuclear architecture (20). This phenomenon is well described in basic cell biological research but has not yet been exploited towards more clinical application. The approach we developed covers this gap and displays the relevant changes as intensity distribution of the two types of signals that reflect said phenomena: (a) 5mC signals created through immunofluorescence targeting using a sandwich assay and (b) gDNA represented by DAPI-signals that are generated by subsequent counterstaining of the same cells, as DAPI intercalates into AT-rich DNA, the main component of highly repetitive and compact heterochromatic sequences. The process results in images that represent maps of interrogated cells with a spectrum of differential DNA methylation phenotypes represented by 5mC/DAPI texture features. One such texture feature that we used to characterize cells is the 5mC/DAPI colocalization index.

Using the 5mC/DAPI index we were able to distinguish between the different cell types based on their differential 5mC/DAPI distribution patterns (scatter plots). In all comparisons between normal and cancerous cells, from cultured cell models over human tissues and epithelial cells derived from patient sputum, we basically saw the same differential 5mC/DAPI distributions and resulting 5mC/DAPI indices. The significant reduction of global 5mC in cancerous cells versus normal epithelial cells, specifically in areas of higher DNA density —delineating more compact genomic regions that predominantly harbor repeat sequences— leads to a shift in the nuclear colocalization of 5mC and gDNA constituted by a lower 5mC/DAPI index. Especially the resemblance between the cell signatures of the more aggressive H157 cells and the hypomethylated N2-type cells found in the sputum of cancer patients and typical tumor tissue cells indicates that the sputum of a cancer patient contains exfoliated epithelial cells of the tumor that can be detected based on aberrant global 5mC content and nuclear distribution.

The results of our analyses are very intriguing and can play a central role in detecting abnormal cells in sputum samples. Nuclear DNA methylation topology may serve as a novel biomarker for the noninvasive detection of malignant cells of the respiratory tract. The fact that epigenetic markers such as 5mC change early in tumor development makes 3D-qDMI in conjunction with noninvasive sputum cytology an attractive approach to be assessed for early lung cancer detection.

### Final conclusions

This study provides proof-of-concept that normal and cancerous cells can be distinguished by their 5mC/DAPI topology as represented by the 5mC/DAPI colocalization index. This method could also differentiate between normal and tumor tissue of the lung and identify exfoliated aberrant cells in the sputum of cancer patients. These results encourage further feasibility and testing of 5mC/DAPI codistribution as a biomarker in noninvasive early lung cancer detection.

## Data availability statement

The raw data supporting the conclusions of this article will be made available by the authors, without undue reservation.

## Ethics statement

The studies involving human participants were reviewed and approved by VA Institutional Review Board. The patients/participants provided their written informed consent to participate in this study.

## Author contributions

JT and FM provided concept and design of the study. FM contributed with providing cultured cells, tissue and sputum samples. JT performed confocal imaging, image analysis, and data analysis with the support of NG. JT wrote the initial draft of the manuscript. FM and NG contributed to manuscript revision and manuscript submission. All authors contributed to the article and approved the submitted version.

## Funding

This study was supported in part by a grant from the Department of Surgery and in part by Technology Ventures (both Cedars-Sinai).

## Acknowledgments

We thank Dr. Gholamhossein Pezeshkpoor (at the time of the study with the Department of Pathology, West Los Angeles VA) for providing deidentified lung tissue.

## Conflict of interest

The Authors have submitted a patent application relevant to the outcome of the study reported in the manuscript.

The authors declare that the research was conducted in the absence of any other commercial or financial relationships that could be construed as a potential conflict of interest.

## Publisher’s note

All claims expressed in this article are solely those of the authors and do not necessarily represent those of their affiliated organizations, or those of the publisher, the editors and the reviewers. Any product that may be evaluated in this article, or claim that may be made by its manufacturer, is not guaranteed or endorsed by the publisher.
